# Sociodemographic and lifestyle factors associated with the Planetary Health diet in the Korean adult population

**DOI:** 10.1371/journal.pone.0345467

**Published:** 2026-04-15

**Authors:** Ujin Lee, Subeen Kim, Eugene Kang, Heejin Lee, Soomin Lee, Jung Eun Lee, Minji Kang

**Affiliations:** 1 Department of Food and Nutrition, Duksung Women’s University, Seoul, Republic of Korea; 2 Department of Nutrition, Harvard T.H. Chan School of Public Health, Boston, Massachusetts, United States of America; 3 Department of Food and Nutrition, Seoul National University, Seoul, Republic of Korea; 4 Research Institute of Human Ecology, Seoul National University, Seoul, Republic of Korea; The Chinese University of Hong Kong, HONG KONG

## Abstract

This study aimed to examine the associations of sociodemographic and lifestyle factors with adherence to the planetary health diet in the Korean adult population. A total of 25,336 participants aged ≥ 19 years who completed a nutrition survey of the 2016–2021 Korea National Health and Nutrition Examination Survey. The planetary health diet score was calculated based on the guidelines set by the EAT-Lancet Commission. Associations between the planetary health diet score and selected sociodemographic and lifestyle factors were assessed using multivariate logistic regression in men and women, separately. The planetary health diet score was dichotomized for men and women based on the median value, and the odds of being greater than the median scores were modeled as a dependent variable. The mean planetary health diet score was significantly higher in women (mean ± se, 9.51 ± 0.01) than in men (9.17 ± 0.02). Older age was associated with the planetary health diet score above the median in both men and women. The associations with marital status and smoking status, prevalence of monthly alcohol use differed by sex. Among men, being widowed and current smokers were associated with having planetary health diet scores below the median, whereas no such associations were observed in women. Women who were never married tended to be in the lower half of the diet score compared with married women, while the prevalence of monthly alcohol use was associated with the diet score above the median, but not in men. Physical activity, dietary supplement use, and body mass index were not significantly associated with the planetary health diet score in both men and women. Sociodemographic and lifestyle factors were associated with the planetary health diet in men and women. These findings may help to identify at-risk populations for nutritional screening and to develop nutritional intervention strategies and educational materials.

## Introduction

Recently, there has been growing global interest in sustainable diets that simultaneously consider human health and environmental preservation. A sustainable diet is defined as one that ensures access to safe and nutritious food for both current and future generations, while minimizing negative environmental impacts and promoting efficient use of resources throughout the food production and consumption processes [1,2]. However, achieving such diets at scale remains challenging, given the rising burden of diet-related health outcomes and the increasing environmental pressures associated with current food systems, including greenhouse gas emissions, land and water use, and biodiversity loss. In response to these global challenges, the EAT-Lancet Commission – comprising international experts – published the report “Food in the Anthropocene” to propose scientific targets for healthy diets from sustainable food systems, aligned with the United Nations Sustainable Development Goals (SDGs) and the Paris Agreement. The report proposed dietary guidelines aimed at promoting both human health and sustainable food production [2]. To promote dietary sustainability, the recommendations emphasize increased intake of plant-based foods such as vegetables, fruits, whole grains, legumes, nuts, and sources of unsaturated fat, while advising a reduction in the consumption of red and processed meats, added sugars, and refined grains [2]. Beyond improving human health, planetary health-aligned diets deliver environmental co-benefits including biodiversity conservation, climate change mitigation, and natural resource conservation – and are increasingly recognized as a foundational element of sustainable food systems.

Building on these recommendations, a planetary health diet index has been developed to assess whether individual dietary patterns align with sustainable diet criteria [2]. Studies using the planetary health diet index have evaluated how diet quality relates to sociodemographic and lifestyle characteristics in diverse populations; in Malmö, Sweden, for instance, women had higher planetary health scores, and higher diet quality was associated with higher education and greater physical activity, lower smoking, and greater consumption of whole grains, fruits, and vegetables [3]. Consistent patterns were observed in a middle-aged Danish cohort: women had higher planetary health diet index scores, and participants with higher scores tended to be more highly educated and more physically active, while reporting lower smoking and lower alcohol intake [4].

While Korean dietary patterns have traditionally emphasized grains and vegetables, sweeping changes accompanied industrialization, rapid economic development, and globalization. Over this period, national data show the macronutrient energy distribution, with carbohydrates, fat, and protein changing from 80.3%: 7.2%: 12.5% in 1969 to 58.2%: 25.9%: 16.0% in 2022, reflecting notable increases in fat and protein and a concomitant decline in carbohydrate [5,6]. Between 2013 and 2022, substantial changes in daily food intake patterns were identified among Korean adults: mean grain consumption declined from 298.5 g to 253.9 g/day and vegetable consumption from 282.2 g to 226.9 g/day, whereas meat increased from 104.4 to 125.0 g/day and beverages from 168.6 to 266.6 g/day [5]. These shifts may have implications for both diet quality and health. Dietary behaviors are socially patterned by sociodemographic characteristics-such as age, sex, educational attainment, and marital status-and by lifestyle factors, including physical activity, smoking, alcohol consumption, and the use of dietary supplements.

This study applies the planetary health diet index to evaluate diet quality among Korean adults and analyzes its associations with major sociodemographic and lifestyle factors, providing foundational evidence for public health and sustainable diet policies in Korea.

## Materials and methods

### Study design and participants

This cross-sectional study analyzed data from the 2016–2021 Korea National Health and Nutrition Examination Survey (KNHANES) and is reported in accordance with the STROBE (Strengthening the Reporting of Observational Studies in Epidemiology) guidelines (https://www.strobe-statement.org/). We included adults aged 19 years or more who completed all three survey components (health interview, health examination, and nutrition survey) and excluded those with a self-reported history of stroke, myocardial infarction or angina, diabetes mellitus, or major cancers (stomach, liver, colorectal, breast, cervical, lung, or thyroid) (n = 4,476), those missing anthropometric or biochemical measurements (n = 2,513), and pregnant participants (n = 78); the final analytic sample comprised 25,336 individuals (men: 10,630; women: 14,706) [7]. Written informed consent was obtained from all participants by the KNHANES investigators prior to survey participation. The KNHANES protocol was reviewed and approved by the Institutional Review Board of the Korea Centers for Disease Control and Prevention (IRB approval numbers: 2018-01-03-P-A, 2018-01-03-C-A, 2018-01-03-2C-A, 2018-01-03-5C-A).

### Planetary health diet score calculation

Dietary intake was assessed using a one-day 24-hour dietary recall collected in the KNHANES dietary survey. Respondents reported all foods and beverages consumed on the day preceding the interview; for each eating occasion, interviewers recorded meal type, time, location, whether the meal was consumed at home or obtained outside the home, whether it was eaten alone or with others, and the name and amount of each item. The adherence to the planetary health recommendations was operationalized using the Oxford cohort of the European Prospective Investigation into Cancer and Nutrition (EPIC-Oxford) EAT-Lancet diet score, which translates the Commission’s recommendations into a quantitative index; points were assigned to the predefined components and summed to yield an overall score [8]. Adherence to the planetary health diet was assessed with a 14-component score, following the EPIC-Oxford operationalization. For each component, 1 point was assigned when the intake met the component-specific criterion and 0 otherwise; points were summed to yield a total score ranging from 0 to 14, with higher values indicating greater adherence. The component cut-offs were: (1) grains (e.g., rice, wheat products, maize), ≤ 464 g/day; (2) root and starch vegetables (e.g., potatoes), ≤ 100 g/day; (3) vegetables, ≥ 200 g/day; (4) fruits, ≥ 100 g/day; (5) dairy (milk and dairy products), ≤ 500 g/day; (6) red meat (beef, lamb, pork), ≤ 28 g/day; (7) poultry, ≤ 58 g/day; (8) eggs, ≤ 25 g/day; (9) fish, ≤ 100 g/day; (10) legumes (dried beans, lentils, peas), ≤ 100 g/day; (11) soy foods (e.g., tofu, soy milk), ≤ 50 g/day; (12) nuts (peanuts and tree nuts), ≥ 25 g/day; (13) added fats, operationalized as an unsaturated-to-saturated fatty acid ratio ≥ 0.8 (e.g., palm oil, unsaturated fats, dairy fat, lard); and (14) added sugars (sugars and caloric sweeteners), ≤ 31 g/day. For analytical purposes, the total planetary health diet score was categorized by dichotomizing at the sex-specific median of the score distribution in the study population. Participants with a total score ≥ the sex-specific median were classified as having higher adherence, whereas those with a score < the median were classified as having lower adherence.

### Sociodemographic and lifestyle characteristics

Sociodemographic and lifestyle factors included age, marital status, smoking status, monthly alcohol consumption, physical activity, body mass index (BMI), and dietary supplement use. Marital status was categorized as married, separated/divorced, widowed, or never married. Smoking status was classified as never smoker, former smoker, or current smoker. Monthly alcohol consumption was defined as consuming ≥ 1 drink per month during the past year (drinkers) versus lifetime abstainers or those consuming < 1 drink per month in the past year (non-drinkers). Physical activity was classified as sufficiently active if participants reported ≥ 150 minutes/week of moderate-intensity activity or ≥ 75 minutes/week of vigorous-intensity activity, or an equivalent combination in which 1 minute of vigorous activity counted as 2 minutes of moderate activity; otherwise, participants were classified as insufficiently active. BMI was calculated from directly measured height and weight as weight (kg)/height (m)^2^ and classified into three categories: < 18.5, 18.5 ≤ BMI < 25.0, and ≥ 25.0 kg/m^2^. Dietary supplement use was defined as use for at least 2 weeks during the past year (users vs. non-users).

### Statistical analysis

All analyses accounted for the complex sampling design of KNHANES by incorporating sampling weights, stratification, and primary sampling units. Analyses were conducted separately for men and women. This sex-stratified approach was prespecified because dietary behaviors and lifestyle characteristics differ by sex, and sex may modify the associations between sociodemographic/lifestyle factors and adherence to a planetary health diet. Continuous variables are presented as survey weighted means and standard errors (SEs), and categorical variables as survey weighted proportions with SEs. Differences by sex were assessed using survey weighted t-test for continuous variables and the Rao-Scott chi-square test for categorical variables.

Associations between the planetary health diet score and sociodemographic and lifestyle factors were examined using survey weighted multivariable logistic regression model. The total planetary health score was dichotomized at the sex-specific median and modeled as the dependent variable. In multivariable models, we adjusted for the following covariates: age group (19–39, 40–64, ≥ 65 years), marital status (married, separated/divorced, widowed, never married), smoking status (never, former, current), monthly alcohol consumption (yes/no), physical activity (yes/no), BMI (< 18.5, 18.5 ≤ BMI < 25.0, ≥ 25.0 kg/m^2^), dietary supplement use (yes/no). For sociodemographic and lifestyle covariates, missing values were imputed using the variable-specific mode. The proportion of missing values was < 5.4% for all variables (marital status: n = 6, smoking status: n = 207; monthly alcohol consumption: n = 193; physical activity: n = 1,301; dietary supplement use: n = 1). All statistical analyses were performed using SAS 9.4 (Statistical Analysis System, SAS Institute, NC, USA). Statistical significance was defined as two-sided p < 0.05.

## Results

### Sociodemographic and lifestyle characteristics

Participants included 10,630 men and 14,706 women; the weighted mean age was 44.7 years for men and 46.8 years for women (Table 1). Among marital status categories, the proportion married was 63.0% in men and 64.9% in women; widowhood was more common among women, whereas never-married status was more common among men. For smoking, the share of never smokers was 32.6% in men versus 89.1% in women, and current smoking prevalence was 33.8% in men compared with 5.8% in women. The proportion reporting ≥ 1 drink per month was 71.5% in men and 46.8% in women, indicating greater alcohol use among men. Physical inactivity was higher in women (57.5%) than in men (51.4%). The prevalence of obesity was higher in men (42.6%) than in women (26.3%), whereas normal weight was more common among women (men 54.2% vs women 67.4%). Dietary supplement use was also higher among women than men (men 52% vs women 62.1%).

**Table 1:** Characteristics of study participants in the planetary health diet.

Characteristics	Men (n = 10,630)	Women (n = 14,706)
**Age (years)**	44.7 ± 0.2	46.8 ± 0.2
**Energy intake (kcal)**	2,342.1 ± 12.3	1,647.4 ± 7.4
**Age group**		
19-39	41.2 (0.7)	36.0 (0.5)
40-64	46.1 (0.6)	48.1 (0.5)
≥ 65	12.7 (0.4)	15.9 (0.4)
**Marital status**		
Married	63.0 (0.7)	64.9 (0.5)
Separated/divorced	3.6 (0.2)	5.1 (0.2)
Widowed	1.2 (0.1)	8.8 (0.3)
Never married	32.2 (0.7)	21.2 (0.5)
**Smoking status**		
Never smokers	32.6 (0.5)	89.1 (0.3)
Former-smokers	33.6 (0.5)	5.1 (0.2)
Current smokers	33.8 (0.6)	5.8 (0.3)
**Prevalence of monthly alcohol use (≥1/month)**		
Yes	71.5 (0.5)	46.8 (0.5)
No	28.5 (0.5)	53.2 (0.5)
**Physical activity** ^a^		
Yes	48.6 (0.6)	42.5 (0.5)
No	51.4 (0.6)	57.5 (0.5)
**Body mass index (kg/m**^**2**^)		
BMI < 18.5	2.8 (0.2)	6.3 (0.3)
18.5 ≤ BMI < 25	54.2 (0.6)	67.4 (0.5)
25 ≤ BMI	42.6 (0.6)	26.3 (0.5)
**Dietary supplement use**		
Yes	52.0 (0.6)	62.1 (0.5)
No	48.0 (0.6)	37.9 (0.5)

Values are presented as mean ± SE or row percents (%, SE).

^a^Physical activity: Individuals who engaged in at least 2 hours and 30 minutes of moderate-intensity physical activity, or 1 hour and 15 minutes of vigorous-intensity physical activity, or an equivalent combination of both (with 1 minute of vigorous activity corresponding to 2 minutes of moderate activity) per week.

### Adherence to the planetary health diet

As shown in Table 2, the mean planetary health diet score was 9.2 points in men and 9.5 points in women, with women scoring significantly higher (p < 0.001). When assessed against the planetary health component-specific cut-offs, adherence to the grains criterion was 36.8% in men and 27.6% in women-higher in men, but below 50% in both sexes. Adherence to the root and starchy-vegetable criterion was high in both sexes (men: 90.4%; women: 89.0%). For vegetables, adherence was higher in men (men: 73.1%; women: 58.4%), whereas for fruits it was higher in women (men: 36.2%; women: 47.0%). The dairy criterion was met by ≥ 98% of both sexes. For animal-source protein components, adherence tended to be higher among women-red meat (men: 31.0% vs women: 45.6%), poultry (men: 81.9% vs women: 87.3%), and fish (men: 59.1% vs women: 69.7%). The legumes and soy-foods criteria were met by ≥84% in both sexes. In contrast, adherence to the nuts criterion was low (men: 4.4%; women: 4.3%) and did not differ significantly by sex (p ≥ 0.05). The added-fat (unsaturated-to-saturated fatty acid ratio) criterion was met by 97.7% of men and 96.8% of women, and adherence to the added-sugar criterion was higher in women than in men (men: 64.7% vs women: 75.7%). Overall, women showed higher total planetary health scores than men, particularly for fruit, red meat, fish, and added sugars, whereas men more often met the grains and vegetable criteria.

**Table 2:** Proportion of participants adhering to the planetary health diet score recommendations.

Dietary components	Criteria for scoring 1 point	Men (n = 10,630)	Women (n = 14,706)	P-value
Planetary health diet score	range 0–14 points	9.17 (0.02)	9.51 (0.01)	<.0001
Whole grains				
1. Rice, wheat, corn, and other	≤ 464 g/day and whole grain fiber > 5 g	36.8 (0.6)	27.6 (0.5)	<.0001
Tubers and starchy vegetables				
2. Potatoes and cassava	≤ 100 g/day	90.4 (0.4)	89.0 (0.3)	0.0028
Vegetables				
3. All vegetables	≥ 200 g/day	73.1 (0.5)	58.4 (0.5)	<.0001
Fruits				
4. All fruits	≥ 100 g/day	36.2 (0.6)	47.0 (0.6)	<.0001
Dairy foods				
5. Whole milk or derivative equivalents	≤ 500 g/day	98.4 (0.2)	98.8 (0.1)	0.0405
Protein sources				
6. Beef, lamb, pork	≤ 28 g/day	31.0 (0.5)	45.6 (0.5)	<.0001
7. Chicken, other poultry	≤ 58 g/day	81.9 (0.4)	87.3 (0.3)	<.0001
8. Eggs	≤ 25 g/day	59.8 (0.6)	63.0 (0.5)	<.0001
9. Fish	≤ 100 g/day	59.1 (0.6)	69.7 (0.5)	<.0001
Legumes				
10. Dry beans, lentils, peas	≤ 100 g/day	99.9 (0.03)	99.9 (0.03)	0.5015
11. Soy foods	≤ 50g/day	84.1 (0.4)	87.5 (0.3)	<.0001
12. Peanuts or tree nuts	≥ 25 g/day	4.4 (0.2)	4.3 (0.2)	0.7446
Added fats				
13. Palm oil, unsaturated oils, dairy fats (incl. in milk), lard or tallow	Ratio of 0.8 for unsaturated: saturated fat intake	97.7 (0.2)	96.8 (0.2)	<.0001
Added sugars				
14. All sweeteners	≤ 31 g/day	64.7 (0.6)	75.7 (0.5)	<.0001

Values are presented as row percents (%, SE).

P-values are based on the Rao-Scott Chi-Square Test.

### Planetary health score distribution by sex

**Fig 1:**
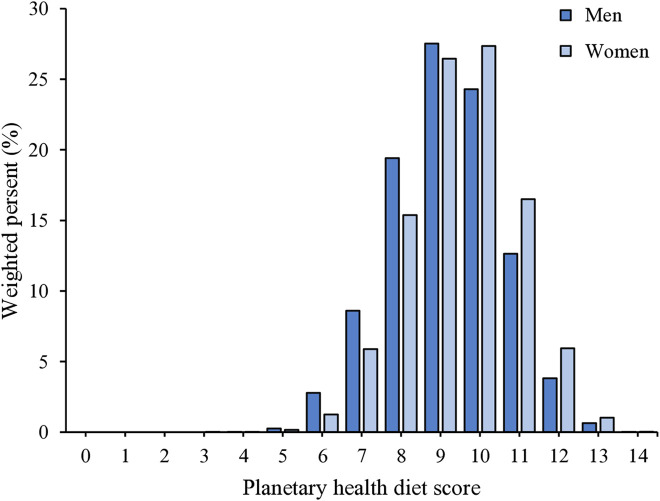
Distribution of planetary health diet scores according to sex.

In sex-stratified analyses, the distribution of total planetary health scores peaked at 9 points in men and 10 points in women (Fig 1). Women exhibited a distribution shifted toward higher planetary health scores compared with men. Specifically, the proportion of individuals achieving scores of 10 points (27.3% women vs. 24.3% men), 11 points (16.5% vs 12.6%), and 12 points (5.9% vs 3.8%) was higher in women, indicating a greater concentration of higher scores among men.

### Associations between planetary health diet score and sociodemographic/lifestyle factors

Older age was associated with greater odds of adherence to the higher planetary health diet (Table 3). Compared with ages 19−39 years, the odds of high higher planetary health adherence in those ≥ 65 years were 6.52 (95% confidence interval (CI): 5.36–7.93) and 3.11 (95% CI: 2.69–3.60) in men and women, respectively, suggesting a stronger association in men. Among women, never-married status was associated with lower odds of high higher planetary health adherence (vs married: Odds ratio (OR) = 0.75, 95% CI: 0.66–0.85). In men, widowed participants also had lower odds of high adherence compared with married men (OR = 0.61; 95% CI: 0.37–0.99), indicating that marital status relates to adherence in both sexes. Current smoking in men was associated with lower odds of high adherence compared with never smoking (OR = 0.81; 95% CI: 0.72–0.92); this association was not significant in women. Among women, non-drinkers had higher odds of high adherence than monthly drinkers (OR = 1.15; 95% CI: 1.06–1.25), whereas alcohol use was not significantly associated with adherence in men. Physical activity, BMI category, and dietary supplement use were not significantly associated with higher planetary health adherence in either sex.

**Table 3:** Odds ratios and 95% confidence intervals from multivariate analyses of sociodemographic and lifestyle factors associated with higher planetary health diet score.

Characteristics	Men (n = 10,630)	Women (n = 14,706)
**Age group**		
19 - 39	1.00	1.00
40 - 64	1.96 (1.73-2.23)	1.98 (1.78-2.19)
≥ 65	6.52 (5.36-7.93)	3.11 (2.69-3.60)
**Marital status**		
Married	1.00	1.00
Separated/divorced	0.93 (0.72-1.22)	1.10 (0.92-1.31)
Widowed	0.61 (0.37-0.99)	1.10 (0.96-1.27)
Never married	1.01 (0.89-1.15)	0.75 (0.66-0.85)
**Smoking status**		
Never smokers	1.00	1.00
Former smokers	1.02 (0.89-1.17)	0.94 (0.78-1.12)
Current smokers	0.81 (0.72-0.92)	0.85 (0.70-1.02)
**Prevalence of monthly alcohol use (≥ 1/month)**		
Yes	1.00	1.00
No	1.02 (0.92-1.14)	1.15 (1.06-1.25)
**Physical activity** ^a^		
Yes	1.00	1.00
No	0.98 (0.89-1.09)	0.99 (0.92-1.08)
**Body mass index (kg/m**^**2**^)		
BMI < 18.5	0.85 (0.63-1.14)	1.17 (0.98-1.40)
18.5 ≤ BMI < 25	1.00	1.00
25 ≤ BMI	0.97 (0.87-1.07)	1.05 (0.96-1.15)
**Dietary supplement use**		
Yes	1.00	1.00
No	0.95 (0.86-1.05)	0.98 (0.90-1.06)

The odds ratio, along with the 95% confidence interval, for scores greater than the median.

All odds ratios were estimated using multivariable logistic regression adjusted for age, marital status, smoking status, prevalence of monthly alcohol use, physical activity, body mass index and dietary supplement use.

^a^Physical activity: Individuals who engaged in at least 2 hours and 30 minutes of moderate-intensity physical activity, or 1 hour and 15 minutes of vigorous-intensity physical activity, or an equivalent combination of both (with 1 minute of vigorous activity corresponding to 2 minutes of moderate activity) per week.

## Discussion

In this study of Korean adults, we examined associations between the higher planetary health diet score and sociodemographic and lifestyle factors. On average, women had higher total planetary health scores than men, driven by greater adherence to the component-specific cut-offs for fruit, red meat, fish, and added sugars. By contrast, men more often met the grains and vegetables criteria. Older age was associated with greater odds of higher planetary health adherence, and adherence varied by marital status. Specifically, widowed men and never-married women were more likely to have lower diet quality (i.e., lower adherence) than their married counterparts. Among men, current smoking was associated with lower adherence compared with never smoking, whereas among women, abstainers had higher adherence than monthly drinkers. In contrast, physical activity, BMI, and dietary supplement use showed no significant associations with adherence in either sex.

Using the higher planetary health diet score as a proxy for sustainable dietary patterns, we examined sociodemographic and lifestyle correlates among Korean adults. Our findings are consistent with studies conducted in Korea and other countries that assessed overall diet quality using various indices-including the Korea Healthy Eating Index (KHEI), Healthy Eating Index (HEI), and alternate Mediterranean Diet (aMED) [3,4,9–14]. In Korea study using the KHEI, overall diet quality was higher among women, older adults, and those with higher educational attainment and income, whereas it was lower among current smokers, individuals who drank at least monthly, and those with obesity [9]. This pattern is consistent with evidence from the United States, Australia, and Brazil: older age, higher educational attainment, greater physical activity, and multivitamin use are positively associated with overall diet quality, whereas overweight/obesity, cigarette smoking, and heavy alcohol consumption are inversely associated [3,4,11–16]. For example, in a large study of multiethnic population, older age, higher educational attainment, greater physical activity, and multivitamin use were significantly associated with having diet quality scores above the median, whereas cigarette smoking and alcohol consumption were associated with below-median scores on the HEI-2010, aHEI-2010, and the aMED score [13].

We also found that associations between sociodemographic and lifestyle factors and diet quality (planetary health adherence) differed by sex. Notably, the positive gradient with age was evident in both men and women, with older age associated with better diet quality, aligning with prior studies [11,12,17,18]. The positive age gradient observed in this study may reflect that older adults are more likely to maintain traditional dietary patterns, whereas younger adults are more frequently exposed to food environments characterized by higher levels of eating out and processed food consumption, which may in turn contribute to lower adherence [19,20]. In contrast, smoking, alcohol use, and marital status exhibited sex-specific patterns. In our study, male current smokers were more likely than never smokers to have higher planetary health diet scores below the sex-specific median (i.e., lower diet quality), whereas no significant difference by smoking status was observed in women. For alcohol, women who drank had significantly lower diet quality than non-drinkers, while no association was detected in men. These sex-specific findings are consonant with evidence from Spain, where high-risk alcohol consumption was associated with lower adherence to the Mediterranean dietary pattern in both men (OR = 1.84, 95% CI: 1.49–2.28) and women (OR = 1.42, 95% CI: 1.19–1.70), with a larger magnitude in men [21]. Marital status also showed sex-specific differences: widowed men had a higher likelihood of lower diet quality (i.e., lower planetary health adherence), whereas never-married women were more likely to have lower diet quality than married women. These patterns are consistent with findings from a large multiethnic study, which showed that men—but not women—who were widowed, underweight, or former smokers were more likely to be in the lower half of the HEI-2010 and aMED distributions [13]. Differences by marital status may reflect variations in the eating context, such as whether one lives with family members, the burden of meal preparation, levels of social support, and the frequency of eating alone. In particular, widowed men may be more vulnerable in terms of meal preparation capacity and dietary self-management within the context of traditional gendered divisions of household labor [22], implying that social circumstances affect diet differently in men and women.

Meanwhile, women had significantly higher total planetary health diet scores than men, and their scores were more concentrated in the upper range (10–12 points). This suggests that the sex gap in adherence is evident not only in mean differences but also across the overall score distribution. Notably, compliance patterns differed by sex across dietary components: men showed relatively higher compliance with the grains and vegetables criteria, whereas women had higher compliance with the fruit criterion. This finding is consistent with a national survey data based KHEI analysis reporting that men score higher on total vegetable intake but lower than women on total fruit/fresh fruit intake [9]. These differences may also reflect sex-specific social and behavioral factors that shape dietary choices. Evidence that women tend to have higher nutrition knowledge and overall diet quality [23] suggests that stronger social expectations for healthy eating and self-management norms among women may facilitate the accumulation of nutrition-related knowledge and translate into healthier dietary choices in practice. Taken together, strategies to improve adherence to planetary health diets should be differentiated by sex, prioritizing not only the components in which each group shows relative need for improvement but also the socio-cultural perceptions and norms that shape these patterns.

To evaluate sustainable dietary patterns, we used the planetary health diet score, which is constructed from the EAT-Lancet Commission’s recommendations that jointly address human health and environmental sustainability. We followed the EPIC-Oxford operationalization, which translates these recommendations into a 14-component binary index (1 point when the component-specific intake meets the recommended level; 0 otherwise) [8]. The planetary health diet score has been applied across diverse countries and, in several cases, adapted to reflect national dietary patterns and cultural contexts by modifying component definitions or scoring schemes. For example, Brazil’s Planetary Health Diet Index and a U.S. adaptation address the limitations of a purely dichotomous scoring scheme by assigning proportional scores (0–10) to each component and summing them to a total score, thereby providing a more granular assessment of adherence to sustainable diets [24,25].

In parallel, Korea has developed assessment tools that reflect national dietary habits to evaluate overall diet quality-most prominently the KHEI [26], Nutrition Quotient (NQ) [27–30], and the Korean Dietary Quality Index [31]. These instruments provide a comprehensive assessment of diet quality among Koreans by organizing items into conceptual domains, typically adequacy, moderation, and balance (or, in several indices, balance, moderation, and practice). The KHEI was developed in 2015 based on the U.S. HEI and adapted to Korean dietary practices. The KHEI comprises 14 components: eight adequacy items (breakfast frequency; whole/mixed grains; total fruit; fresh fruit; total vegetables; vegetables excluding kimchi; meat, fish, eggs, and legumes; and frequency of milk/dairy consumption), three moderation items (percentage of energy from saturated fat; sodium intake; percentage of energy from sweets and beverages), and three balance items (energy intake adequacy; percentage of energy from carbohydrates; percentage of energy from fat). The total score ranges from 0 to 100 [26]. The life-cycle Nutrition Quotient includes age-specific instruments; the adult NQ rates overall diet quality and behaviors via 18 checklist items across balance, moderation, and practice domains, with item scores (0–100) weighted and summed [27–30]. However, these indices are primarily nutrition-focused and do not explicitly incorporate the environmental impacts of diets; accordingly, there is a need in Korea to develop a Korean-specific sustainable diet index that reflects local food culture and consumption patterns while integrating environmental sustainability considerations.

This study has several limitations. First, dietary intake was assessed using a single 24-hour dietary recall for the day preceding the interview; therefore, these data may not reflect usual intake and are subject to day-to-day variation and reporting error. Second, because this analysis is based on cross-sectional KNHANES data, temporal ordering cannot be established and the observed associations between the planetary health diet score and sociodemographic or lifestyle factors cannot be interpreted as causal. Third, we did not specifically account for lactation status, and dietary needs during lactation differ from those of the general population; thus findings should be interpreted cautiously for lactating women.

## Conclusions

In this study of Korean adults, we examined how adherence to the EAT-Lancet diet-a proxy for sustainable diet quality-relates to sociodemographic and lifestyle characteristics. Adherence was associated with age, marital status, smoking, and alcohol use, and these associations differed by sex; diet quality increased with age in both sexes; among women, non-drinkers tended to have higher adherence; among men, widowhood and current smoking were linked to lower adherence; and among women, never-married status was associated with lower adherence. These findings suggest the need for sex- and socially tailored nutrition education and intervention strategies. The findings also highlight that the EAT-Lancet score may be useful for assessing sustainable dietary patterns in Korea and for informing population monitoring and nutrition policy development.

## Abbreviations

BMI: body mass index
CI: confidence interval
EPIC: Oxford European Prospective Investigation into Cancer and Nutrition-Oxford
HEI: Healthy Eating Index
KNHANES: Korean National Health and Nutrition Examination Survey
KHEI: Korea Healthy Eating Index
OR: odds ratio
SE: standard error.
